# The effect of objectifying media images on eating pathology: an experimental study comparing Australian and Asian females

**DOI:** 10.1186/2050-2974-3-S1-P6

**Published:** 2015-11-23

**Authors:** Charmain Tan, Victoria Yeung, Tara de Paoli, Stephen Loughnan, Isabel Krug

**Affiliations:** 1 University of Melbourne, Melbourne, Australia; 2 The Chinese University of Hong Kong, Hong Kong, Hong Kong; 3 University of Edinburgh, Edinburgh, Scotland, UK

## Introduction

The onset of eating pathology has commonly been attributed to media influences. However, most of these studies have not included an experimental design and have mainly concentrated on Caucasian samples, with limited research on non-Western populations.

## Objective

To assess whether exposure to either objectifying female media images or neutral images of objects (e.g. chairs) had an impact on eating pathology and self-objectification and whether this effect was different for Australian and Asian females.

## Method

A total sample of 301 female participants [Caucasian Australians (n= 97); Asians grown up in Australia (n = 70), Asians currently residing in Australia (n = 60) and Chinese living in Hong Kong (n = 74)] were exposed to a slideshow of either objectifying women (n=147) or neutral (n=154) images. Variables associated with the objectification theory framework and disordered eating attitudes and symptoms were assessed through self-report.

## Results

State self-objectification was higher in individuals who were exposed to the objectifying media images, regardless of ethnicity (p <0.01). Caucasians had significantly higher BMI and greater body surveillance compared to the Chinese population (p<0.01), and more trait self-objectification and body surveillance compared to Asians residing in Australia (p<0.05). Similarly, Asians who grew up in Australia demonstrated higher trait self-objectification compared to Asians residing in Australia (p<0.05), and body surveillance and food preoccupation compared to the Chinese sample (p<0.05).

## Conclusions

The results indicated that self-objectification can be elicited from exposure to objectifying media images in women from varying cultural backgrounds. This understanding is crucial to the development of preventive measures of eating pathology.

